# Establishing a Clinical Trial Quality Team in a Comprehensive Cancer Center: A Strategy to Navigate the New European Regulatory Landscape

**DOI:** 10.3390/curroncol33070418

**Published:** 2026-07-11

**Authors:** Francesco Callegarin, Elisa Masetto, Beatrice Basaldella, Paola Del Bianco, Giulia Doria, Denise Kilmartin, Giovanna Magni, Giacomo Moratello, Giorgia Pagan, Angela Paggio, Lisa Perilli, Paola Rescigno, Gian Luca De Salvo

**Affiliations:** Clinical Research Unit, Veneto Institute of Oncology IOV—IRCCS, 35128 Padua, Italy; francesco.callegarin@iov.veneto.it (F.C.); elisa.masetto@iov.veneto.it (E.M.); beatrice.basaldella@iov.veneto.it (B.B.); paola.delbianco@iov.veneto.it (P.D.B.); giulia.doria@iov.veneto.it (G.D.); denise.kilmartin@iov.veneto.it (D.K.); giovanna.magni@iov.veneto.it (G.M.); giacomo.moratello@iov.veneto.it (G.M.); giorgia.pagan@iov.veneto.it (G.P.); angela.paggio@iov.veneto.it (A.P.); lisa.perilli@iov.veneto.it (L.P.); paola.rescigno@iov.veneto.it (P.R.)

**Keywords:** CTQT, oncology, clinical trial, quality, non-profit, URC, KPIs

## Abstract

Background: Clinical research has evolved into a multidisciplinary field integrating Medical Devices (MD), In Vitro Diagnostics (IVD), and Artificial Intelligence (AI), governed by a modernized European regulatory framework including the Clinical Trial Regulation (CTR), the Medical Device Regulation (MDR), and the In Vitro Diagnostics Regulation (IVDR). In 2021, the Istituto Oncologico Veneto (IOV) IRCCS established the Clinical Trial Quality Team (CTQT) to provide support for non-profit trials and ensure high-quality standards. Methods: A descriptive analysis of trials managed between 2021 and 2025 was conducted using the REDCap platform. A team of 15 professionals assessed performance via Key Performance Indicators (KPIs) categorized into: ethical–regulatory compliance, operational efficiency and data quality. Results: The CTQT manages 18 studies (83% interventional, 50% multicentre), primarily focused on brain (17%) and genitourinary (23%) tumours. The mean time from internal feasibility to Ethics Committee discussion is 13 days. Total approval time (ethical and administrative) is 107 days. Operational metrics are strong, with Site Initiation Time averaging 27 days and First Patient In (FPI) at 41 days. Conclusions: A centralized, multidisciplinary structure effectively supports non-profit research in a complex regulatory environment. While operational speed is high, challenges such as staff turnover and query management variability remain. Future efforts will focus on standardizing post-approval administrative phases and optimizing data management to further improve trial efficiency and integrity.

## Simple Summary

To address the evolving European regulatory landscape, the Veneto Institute of Oncology established the Clinical Trial Quality Team to provide specialized support for non-profit clinical research. This study evaluates the team’s performance between 2021 and 2025, aiming to demonstrate how a centralized, multidisciplinary approach ensures high quality and efficiency in investigator-initiated trials. By analyzing key performance metrics, the authors show that this model achieves rapid trial activation and enrollment times, often outperforming international benchmarks. These findings provide the research community with a scalable organizational framework to manage complex regulations while maintaining data integrity. Ultimately, this model highlights the importance of dedicated professional roles in sustaining high-quality academic research in oncology.

## 1. Introduction

Clinical research has transitioned from its conventional paradigms into a highly multidisciplinary field that now integrates Medical Devices (MD), In Vitro Diagnostics (IVD), Artificial Intelligence (AI), and Advanced Therapy Medicinal Products (ATMP). This transformation is driven by a modernized European regulatory framework, including Regulation (EU) 536/2014 (CTR) [1], the Medical Device Regulation 745/2017 (MDR) [2] and the In Vitro Diagnostics Regulation 746/2017 (IVDR) [3], designed to enhance safety and efficacy standards across all areas. Regulation (EU) 536/2014 (CTR) has transformed European clinical research by introducing “low-intervention” trials and a risk-based management approach. The transition to the Clinical Trial Information System (CTIS) for submissions remains a significant operational challenge for both commercial and non-profit sponsors [1]. Consequently, a significant shift has occurred in the clinical trial landscape, requiring stakeholders to navigate new regulatory domains, including those for MD and in vitro diagnostic medical devices (IVDs). These areas are governed at the European level by MDR and IVDR, which repealed the previous Directives.

These changes coincided with the long-awaited revision of the ICH E6 (R2) guidelines, better known as Good Clinical Practice (GCP), which was updated to the ICH E6 (R3) [4] version that came into force in July 2025. This update introduced significant innovations and emphasized several key areas of interest, necessitating a comprehensive training update for both sponsors and clinical centres. In this evolving landscape, non-profit clinical research plays a pivotal role. As these investigator-initiated studies are primarily driven by academic and public institutions rather than commercial interests, they address critical clinical gaps and rare diseases. Nonetheless, they remain subject to the same stringent regulatory requirements as commercial trials, demanding high standards of GCP and rigorous data integrity. The introduction of the aforementioned regulations and updates has led to a clear paradigm shift, introducing significant innovations not only in the regulatory field, but also in numerous technical–scientific aspects. This has translated and still translates into a need for Italy to adopt new organizational methods, as well as new technical–scientific and administrative profiles. The IOV (Istituto Oncologico Veneto) is the first public institute in the Veneto region dedicated to comprehensive cancer care and research. Recognized as an IRCCS since 2005 and designated as a Regional Reference Hub, all IOV locations have together been OECI-accredited as a Comprehensive Cancer Center since 2021. The Clinical Research Unit (CRU) manages both internally and externally sponsored research through a multidisciplinary team providing end-to-end support, from study design and biostatistics to regulatory and administrative management. The Clinical Research Coordinator (CRC) holds a central role within the unit, with specialists currently operating across various oncology areas. The CRU is organized into three specialized offices: Biostatistics and Bioinformatics; Regulatory Procedures, including legal, administrative, and technical–scientific profiles; and a Clinical Trials Center (CTC), which is dedicated to the operational management of studies.

In response to the need to conduct non-profit Phase I clinical trials, the IOV self-certified its status as a Phase I center (AIFA Resolution n. 809/2015) [5] and established the Clinical Trial Quality Team (CTQT) through resolution no. 820 dated 29 October 2021. The team operates within the CRU to maintain high-quality regulatory standards.

While the CRU previously managed non-profit research, the evolving regulatory landscape necessitated a structural transition. Specifically, the establishment of the CTQT became essential for conducting Phase I non-profit trials, ensuring full compliance with the stringent quality and safety standards mandated by AIFA Resolution 809/2015.

The primary role of the CTQT is to support IOV researchers in the design, initiation, conduct and conclusion of non-profit clinical trials with the IOV as a sponsor, ensuring compliance with quality standards and current regulations (GCP).

The main tasks of the CTQT include:

- Assistance in the drafting of protocols, dossiers, and other essential documents for submission to regulatory authorities and Ethics Committees (ECs).
- Collaboration in study design, sample size calculation, electronic database creation, and data quality management.
- Managing budgeting, liaison with competent authorities, assisting in drug management and pharmacovigilance, and planning and conducting study monitoring.

To date, the CTQT has drawn up 18 Standard Operating Procedures (SOPs) and consists of a CTQT Responsible and 15 professionals within its Organizational structure (please see File S1 on Supplementary Material). In particular, the CTQT employs competent personnel who cover the roles of Quality Assurance, Medical Monitor, contact person for regulatory procedures, contact person for the safety of the study drug/device, Clinical Project Manager (CPM), Clinical Monitor, IT Specialist, Statistician, and Administrative Coordinator. Originally established to comply with the stringent requirements of AIFA Resolution 809/2015 for Phase I trials, the CTQT model was strategically extended to all investigator-initiated studies, regardless of phase or design. This expansion was driven by the recognition that the modern regulatory landscape, characterized by the CTR, MDR, and IVDR, imposes a uniform level of complexity and accountability across all clinical research. By centralizing multidisciplinary expertise, the IOV ensures that non-profit research maintains the same quality and data integrity standards as commercial trials, effectively implementing a ‘Quality by Design’ approach aligned with the updated ICH E6 (R3) [4] guidelines.

The workflow for managing a non-profit clinical trial under the proprietary institutional framework developed by the CTQT consists of six main steps designed to standardize local academic operations:

1. Study proposal: The Principal Investigator submits a request via an online form (REDCap survey) or via email. The proposal is then discussed in a periodic meeting.
2. Team assignment: Once the proposal is accepted, a CPM and a dedicated project team are assigned. The responsibilities of each member are defined in the “Project Charter” and the “Study Role”.
3. Planning phase: The planning phase begins with a Kick-off Meeting to define the study design, identify critical issues, and draft the protocol. Under the guidance of the CPM, the study is structured using a Work Breakdown Structure (WBS) and the development of essential strategic documents, specifically the Clinical Project Management Plan (CPMP), Clinical Quality Management Plan (CQMP), Clinical Risk Management Plan (CRMP), and Monitoring Plan. Furthermore, in multicentre studies, site feasibility is assessed via the REDCap platform, using targeted surveys to ensure each centre meets the necessary eligibility and infrastructure requirements.
4. Submission: A person in charge of regulatory procedures sends the document package to the competent authorities (AIFA, EC, Ministry of Health).
5. Study Conduct: Following regulatory approval and administrative resolution, the Clinical Monitor initiates the study with a Site Initiation Visit (SIV) and ensures the quality of the study by means of Interim Monitoring Visits (IMVs).
6. Study closure: Once recruitment and follow-up are complete, individual centres are closed with a “Close Out Visit” (COV). The database is locked and the Clinical Study Report (CSR) is drawn up.

Most of the clinical trials promoted by IOV are conducted with partial financial support from pharmaceutical companies. This is formalized through a “Third Party support” agreement in which the manufacturer contributes financially and provides the investigational product (drug, medical device, etc.) free of charge. Under these agreements, the company retains the right to acquire the study data and results upon completion; however, this option has been exercised in very few cases to date [6,7]. This framework shifts academic research away from the restrictions of older legislation, establishing a virtuous economic cycle where private transferees must reimburse the direct and indirect operational costs incurred by the public sponsor, thereby generating revenue that can be directly reinvested into institutional research infrastructures and patient care [8].

## 2. Materials and Methods

All methods were carried out in accordance with relevant guidelines and regulations. Since this study consists of a retrospective descriptive analysis of aggregated management data and operational performance metrics, it does not involve human participants, clinical interventions, or the use of personal identifiable data. Therefore, formal ethics approval for this specific analysis was not required. Informed consent was obtained from the participants for each study.

This descriptive analysis leveraged existing internal data within the CTQT, made accessible through the centralization of management processes. These processes utilize the REDCap platform for eCRF implementation, data collection, and centralized monitoring activities. The studies were performed in accordance with relevant guidelines and regulations.

While the workflow encompasses six stages, this evaluation focuses on the core operational window, from the feasibility phase to study conduct, where the CTQT’s impact on efficiency, regulatory compliance, and data integrity is most prominent.

The performance of activities was evaluated by identifying a set of Key Performance Indicators (KPIs) categorized into 3 areas of interest: ethical–regulatory compliance, operational indicators and data quality.

Regarding ethical–regulatory compliance, the following indicators are considered:

- The time to EC, defined as the time from feasibility discussion within CRU to EC discussion;
- The time to ethical approval, defined as the time from EC discussion to approval by competent authorities;
- The time to resolution of the study, defined as the time from approval to the final administrative authorization for the conduct of the study.

The following operational indicators were taken into consideration:

- The Site Initiation Time, from the study resolution to the opening of the coordinating centre and the participating centres;
- The time to first randomized patient, from the date of SIV to the first randomized/enrolled patient;
- The screening failure rate, defined as the proportion of screen-failed patients out of the total number of screened patients;
- The ratio of enrolled to expected patients; for ongoing studies, this is calculated as the ratio between the number of patients enrolled and expected as of 31 August 2025. The latter is calculated proportionally considering the expected time of enrolment and the sample size established for the study.

Most of the indicators were selected based on official reports used by other companies to define the performance of individual sponsors and therefore already validated. The remaining indicators were defined to reflect the performance of the CTQT as closely as possible based on the actual reality of the CTQT itself. This multi-dimensional approach to tracking ethical–regulatory, operational, and data quality indicators is strongly supported by the recent literature on multicenter quality tracking. Specifically, contemporary frameworks emphasize that combining yield rates, recruitment velocity, and protocol adherence index metrics allows institutions to objectively quantify site-specific efficiencies and pro-actively deploy data-driven quality management plans [9].

Continuous variables (operational and regulatory timelines) were summarized using medians with standard deviations and medians with interquartile ranges (IQRs) due to the non-normal distribution and skewness of the data. Frequencies and percentages were used for categorical variables. To ensure methodological validity, trials with out-of-sequence operational events (resulting in anomalous negative time intervals) were excluded from aggregate timeline calculations and were instead analyzed qualitatively. All time intervals are reported as whole numbers of days to align with international clinical trial start-up benchmarking standards.

## 3. Results

From 2021 to 2025, the CTQT managed 18 studies divided into interventional clinical trials on drugs, interventional trials without drugs or devices (supplements, foods for special medical purposes, technique), clinical investigations on Post-Marketing Clinical Follow-up (PMCF) medical devices, clinical investigations on pre-market medical devices, clinical investigations on in vitro diagnostic medical devices, Post-Market Performance Follow-up (PMPF), and observational studies. In addition, CTQT follows both single-centre and multi-centre trials. The characteristics of the trials currently managed by CTQT are defined in Table 1.

The CTQT is mainly in charge of interventional studies (83%). Among these, six involve MD and four involve investigational medicinal products (IMPs). Of the drug-related studies, one is phase I, two are phase II, and one is phase III. Nine studies are multicentre, with a minimum of two to a maximum of 19 centres involved. Currently, nine main types of tumours are treated, with a particular focus on brain (17%) and genitourinary (23%) tumours. All studies under the CTQT are currently open, except for one, which was completed in January 2024. A summary of the indicators considered is shown in Table 2.

**Figure 1:**
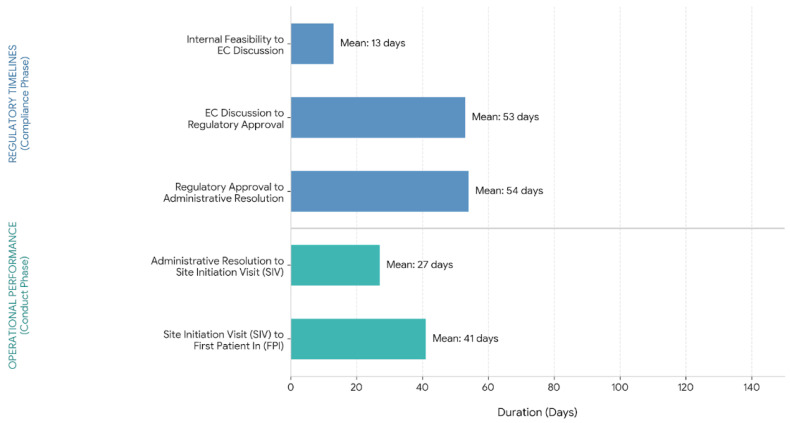
Clinical trial start-up and study activation timelines managed by the CTQT (2021–2025). Horizontal bars represent the mean duration in whole days for each discrete regulatory and operational phase. Out-of-sequence operational events are excluded from the quantitative pooling.

By 31 August 2025, the EC had discussed 14 of the studies under consideration. Of these, 11 had been authorized and the SIV performed, and 10 had started enrolments. Monitoring was performed for seven studies through on-site/remote visits, while query data were available within REDCap for six. The mean time elapsed between the formal feasibility meeting within the CRU and the first discussion of the EC was 13 days. The mean ethical approval time was 53 days, while the mean time between approval and final administrative resolution was 54 days. Combined, these regulatory and compliance phases led to a mean total time of approximately 107 days from the initial EC examination to the final institutional authorization. At the operational level, the mean Site Initiation Time was 27 days. Following site initiation, the mean time to enroll the first patient (FPI) was 41 days. The sequential progression of these clinical trial start-up milestones and their respective distributions are visually summarized in Figure 1.

Out of 10 studies that had started enrolment, four recorded a screening failure rate of more than 10%, with a maximum percentage reached of 19%. In four studies, all screened patients were eligible. For three studies, the number of patients enrolled on 31 August 2025 exceeded their projected interim recruitment targets for that specific time period, demonstrating higher-than-expected enrollment velocity, while for the remaining studies, the percentages of patients enrolled ranged from 0 to 62%. Three out-of-sequence events were observed and analysed separately: two interventional drug trials submitted via the CTIS portal under CTR recorded formal feasibility after EC submission. This occurred because informal clinical feasibility was conducted beforehand, but formal administrative logging was deferred to avoid bureaucratic duplication during the European validation phase. One trial conducted the SIV eight days prior to the formal administrative resolution. This was done to optimize site readiness, though patient recruitment strictly commenced only after the formal resolution was signed. Out of seven monitored studies, the mean number of monitoring visits is 19, with one study reaching 40 visits. The mean number of queries per patient ranges from a minimum of 1.8 to a maximum of 43, while the mean number of days for query resolution ranges from a minimum of 15 days to a maximum of 82 days.

## 4. Discussion

The establishment of the CTQT at the IOV represents a strategic response to the evolving European regulatory landscape (CTR, MDR, and IVDR). Operating within the CRU, this unit ensures that the IOV’s non-profit research, which leads Italy in patient enrolment, upholds the highest GCP quality standards [4]. Originally implemented following AIFA Resolution 809/2019 for Phase I trials [5], CTQT has since expanded its scope to provide comprehensive, end-to-end support for all types of non-profit studies. As of August 2025, the team manages 18 studies (83% experimental), including four involving IMPs, six involving MD, and one involving IVD. The complexity of this portfolio is highlighted by the fact that 50% of these studies are multicentre, involving up to 19 participating sites, necessitating the unit’s specialized multidisciplinary approach. The 18 SOPs, covering all phases of the clinical project, and the centralized use of the REDCap platform for data collection, eCRFs and monitoring, are tools that have been adopted over time to ensure systematization and quality management.

In terms of indicators chosen to evaluate CTQT performance, while our analysis provides a robust descriptive overview of the CTQT’s impact, the necessity for standardized tools in this domain is further emphasized by recent methodological protocols, such as the Clinical Trial Site Performance Measure (CT-SPM) proposed by Bozzetti et al. (2025) [10]. By aligning our operational KPIs with validated psychometric dimensions, specifically in the realms of enrolment efficiency and data quality management, we ensure that the IOV’s internal monitoring systems are consistent with emerging international efforts to quantify site performance and reduce research waste.

As far as Ethical–Regulatory Compliance is concerned, the average time between the internal feasibility discussion and the first discussion of the EC is only 13 days, a figure that suggests rapid internal document preparation. To ensure methodological rigor and avoid data distortion, all out-of-sequence operational events resulting in negative time intervals were excluded from the aggregate statistical calculations. Specifically, two interventional medicinal product trials submitted via the CTIS portal under CTR initially displayed negative intervals when mapping the formal administrative feasibility logging against the EC submission date. A preliminary clinical and scientific feasibility assessment always occurs through informal investigator meetings prior to any regulatory submission. However, under the strict and dynamic CTIS workflow, the formal administrative institutional registration of feasibility was intentionally deferred until the final, approved protocol version was secured. For all other non-CTIS study types, where the regulatory pathway is more linear, the formal feasibility verification remains systematically completed within the CRU before EC submission. This internal step is crucial to certify the centre’s operational capacity and resource availability before formally proceeding with the regulatory submission.

The overall mean time to obtain approval after the discussion of the Territorial Ethics Committee (53 days) and the subsequent resolution (54 days) leads to a mean time of about 107 days from the examination by the RA/EC to the final resolution. In particular, the time between approval and resolution is the most variable indicator, reaching a maximum of 251 days.

At the operational level, a relatively rapid transition from the administrative authorization to the SIV (27 mean days) and a mean time of 41 days from the SIV to the FPI is observed. It should be noted that there is a negative value in the SIV, excluded from the calculation, indicating that the SIV was conducted prior to the formal administrative authorization; the recruitment of patients was initiated only following the resolution.

Regarding ethical approval timelines, the mean time for the CTQT (53 days) was longer than the 2024 benchmarks of 42 days for the European Economic Area (EEA) and 38 days for the United States (US) [11]. In contrast, the mean total time for resolution and SIV (77 days) was substantially faster than the start-up timelines observed in the EEA (339 days) and the US (253 days) [11]. This difference must be contextualized in relation to the timing required by the CTR and submission through the CTIS portal; to date, the CTQT manages only two trials under EU Regulation 536/2014. Overall, the total time between the feasibility date and the SIV averaged 125 days for the CTQT, which is well below the timelines observed in the EEA (416 days) and the US (320 days) [11]. The CTQT performance regarding First Patient In (FPI) was also favorable, showing a median of 41 days compared to an FPI of 67 days in the EEA and 104 days in the US [11]. Importantly, these comparisons between the CTQT experience and broader international metrics are based on different sample sizes, leading to greater variability in local results while pointing to a promising direction in trial management. In particular, the small sample size available at the CTQT leads to greater variability in results, but still points to a promising direction in the management of clinical trials. The lack of a direct national (Italian) benchmark for academic CTQT performance represents a minor challenge in context interpretation. This absence is justified by the fact that institutional, centralized quality teams dedicated exclusively to non-profit research remain highly uncommon within the Italian public healthcare landscape. Most academic centers still rely on decentralized or investigator-driven coordination, making the IOV CTQT model a pioneering framework in Italy and preventing direct peer-to-peer national comparisons.

Results show successful enrolment in some studies, with three studies exceeding their projected interim recruitment targets for that specific time period, demonstrating higher-than-expected enrollment velocity. However, for the remaining studies, enrolment rates range from 0% to 62%, considering that some studies as of 31 August 2025 had only recently been activated. The percentage of screening failure is manageable, with four out of 10 studies recording rates above 10% (maximum 19%).

Data quality monitoring (managed via the REDCap database) reveals intensive monitoring, with a mean of 19 visits per study (with a maximum of 40) across seven studies for which monitoring has been initiated. Despite the high level of monitoring activity, there is a fair amount of heterogeneity in the management of queries. This heterogeneity is manifested in the mean number of queries per patient (from 1.8 to 43), and the mean resolution times (from 15 to 82 days). The variability in query resolution times can be explained by delays in response times from participating centres. A high number of queries and long resolution times suggest that, although monitoring is frequent, there may still be room for improvement regarding the quality of the initial data entered by the participating centres or in the efficiency of the query resolution lifecycle. This operational strain is a well-documented phenomenon; high screen failure rates and excessive paperwork or data-entry burdens significantly exacerbate research coordinator fatigue and accelerate staff turnover [9]. When study personnel experience burnout, it directly translates into clinical site disengagement and delayed query lifecycles, further demonstrating the vital role of a centralized infrastructure like the CTQT in providing continuous administrative mitigation, protocol training, and logistical support to protect trial continuity [9]. The CTQT model provides a solid foundation for continuous quality improvement, aligning the IOV’s non-profit research with the rigorous standards of the updated ICH E6 (R3) guidelines. In fact, the recent update to the ICH E6 (R3) guidelines in July 2025 emphasizes a “Quality by Design” approach and risk-based management [4]. The CTQT is uniquely positioned to lead this transition, as its existing structure already integrates essential strategic documents such as the Clinical Quality Management Plan (CQMP) and Clinical Risk Management Plan (CRMP) during the planning phase. Future development will focus on refining these processes to ensure that quality is built into the protocol from inception, thereby reducing the query burden and improving data integrity. The scalability of the CTQT model lies in its centralized, multidisciplinary architecture and the systematic use of digital platforms like REDCap.

The CTQT is currently composed of 15 specialized professionals, including roles such as CPM, Clinical Monitors and statisticians. However, these internationally recognized professional figures are not yet formally defined within the Italian national administrative framework. Consequently, these specialists are often classified under generic administrative or technical roles that do not accurately reflect their high-level expertise or actual responsibilities. The personnel are funded through a complex synergy of resources, including revenues from commercial (profit) research conducted within the Institute, funding from the Ministry of Health, and co-financing from pharmaceutical companies that provide support for the non-profit studies promoted by the IOV. This reliance on atypical contracts and varied funding streams leads to significant personnel turnover, which threatens the continuity of specialized knowledge. These organizational vulnerabilities are recognized as widespread systemic challenges within the Italian academic clinical environment, which is historically constrained by limited financial resources, heterogeneous internal structures, and a lack of formal profiling for non-investigator research staff within institutional organization charts. To mitigate these deficiencies, the implementation of centralized non-profit research infrastructures, such as the CTQT, becomes paramount, as they provide academic investigators with the standardized quality management systems and operational expertise necessary to achieve the same data integrity standards as commercial trials [8].

This comparison underscores that the integration of specialized expertise, including project management, regulatory oversight, and dedicated monitoring within a centralized unit is a fundamental requirement for ensuring both data integrity and operational efficiency in the non-profit sector. Furthermore, the IOV CTQT structure aligns with the broader ECRIN paradigm, which advocates for harmonized, high-quality management tools to overcome the regulatory fragmentation and operational hurdles inherent to academic sponsor roles [12]. Furthermore, this centralized support framework directly mirrors the operational philosophy of UKCRC-registered Clinical Trials Units, where embedding specialized trial management and methodological infrastructure within dedicated units has proven vital to reduce research waste and standardize study delivery [13]. Our study has some limitations that should be acknowledged. Its retrospective, descriptive design limits the ability to establish causal relationships between specific CTQT interventions and operational metrics. Second, the sample size is relatively small, consisting of 18 trials managed over a four-year period, which naturally introduces higher variability in timelines and performance indicators. Finally, significant heterogeneity exists among the included studies, which span IMPs, MD, IVD and observational designs. While this heterogeneity accurately reflects the real-world portfolio of a comprehensive cancer center navigating the updated European regulatory landscape, it prevents a one-size-fits-all generalizability of the operational metrics.

## 5. Conclusions

The implementation of the CTQT at the IOV represents a successful strategic response to the paradigm shift imposed by the modern European regulatory framework, specifically CTR and the MDR. Our analysis confirms that a centralized, multidisciplinary structure providing end-to-end support, from protocol design to study closure, is essential for maintaining high operational standards in non-profit research.

The centralization of processes, supported by the systematic use of the REDCap platform, has yielded competitive timelines, outperforming recent benchmarks for the EEA and the US, demonstrating that internal efficiency can effectively mitigate external regulatory complexities. The CTQT organization can be exported to other Comprehensive Cancer Centres or academic hubs to harmonize non-profit research standards across different regions. The long-term sustainability and scalability of the CTQT model face a significant structural challenge inherent to the Italian healthcare system. Despite several regulatory hurdles, the IOV has made a profound internal effort to maintain this multidisciplinary unit, representing a model of quality and efficiency that must be continuously implemented.

## Figures and Tables

**Table 1:** Characteristics of the studies currently managed by the CTQT.

Features	N (%)
Type of study	
Observational	2 (11%)
Translational	1 (6%)
Experimental	15 (83%)
Experimental classification	
Drug	4 (27%)
Medical Device	6 (40%)
In Vitro Diagnostics	1 (6.6%)
Food supplement	1 (6.6%)
Technique	1 (6.6%)
Food for special medical purposes	1 (6.6%)
Without drug and device	1 (6.6%)
Multicenter Study	
No	9 (50%)
Yes	9 (50%)
Number of centers	
<10	6 (67%)
≥10	3 (33%)
Tumor type	
Cerebral	3 (17%)
Colon rectum	1 (5.6%)
Leukaemia	1 (5.6%)
Breast	2 (11%)
Pleural/peritoneal mesothelioma	2 (11%)
Lung	1 (5.6%)
Prostate/Bladder	4 (23%)
Sarcoma	1 (5.6%)
Miscellaneous tumors	2 (11%)
Bladder	1 (5.6%)
Status as of 31 August 2025	
Open	17 (94%)
Closed	1 (6%)

**Table 2:** Summary of the indicators considered. Times are expressed in days.

Indicator	N Studies	Min, Max	Median	Mean	IQR	SD	N Studies Excluded Due to Negative Values
Regulatory timelines (Compliance)							
Time from feasibility to EC discussion	14	2, 28	7	13	6–25	31.2	2
Time from EC discussion to approval	11	0, 137	56	53	19–70	45.1	
Time from approval to resolution	11	3, 251	43	54	23–48	67.2	
Recruitment performance (Operational)							
Site Initiation Time	11	0, 75	20	27	6–47	25.8	1
First Patient In (FPI)	10	0, 89	38	41	7–63	30.2	
Monitoring outcomes (Data quality)							
Number of monitoring visits	7	4, 40	17	19	5–37	14.6	

## Data Availability

The data presented in this study are available on request from the corresponding author due to internal policy.

## Supplementary Materials

The following supporting information can be downloaded at https://www.mdpi.com/article/10.3390/curroncol33070418/s1, File S1: CTQT Standard Operating Procedures.

## Abbreviations

The following abbreviations are used in this manuscript:

MD	Medical Device
IVD	In Vitro Diagnostics
CTR	Regulation (EU) 536/2014
CTIS	Clinical Trial Information System
MDR	Regulation (EU) 745/2017
IVDR	Regulation (EU) 746/2017
GCP	Good Clinical Practice
IOV	Istituto Oncologico Veneto
CRU	Clinical Research Unit
CTC	Clinical Trial Center
CTQT	Clinical Trial Quality Team
EC	Ethics Committee
SOPs	Standard Operating Procedures
