# Supplementary material for: Establishing a Clinical Trial Quality Team in a Comprehensive Cancer Center: A Strategy to Navigate the New European Regulatory Landscape
Source: Curr Oncol. 2026 Jul 11;33(7):418. doi: 10.3390/curroncol33070418 (PMC13408362; doi:10.3390/curroncol33070418)
Supplement: Supplementary file 1 [file curroncol-33-00418-s001.zip › curroncol-4336017-supplementary.pdf]

CTQT Standard Operating Procedure

- **I_URC_P01** - STESURA, REVISIONE ED EMENDAMENTI DEL PROTOCOLLO DI STUDIO
- **I_URC_P02** - Stesura e revisione della documentazione per il paziente
- **I_URC_P03** - STESURA DEL CONTENUTO DELLE CRF
- **I_URC_P04** - INVESTIGATOR'S BROCHURE (IB)
- **I_URC_P05** - Documentazione da inviare al CE e AC per l'approvazione del protocollo e/o eme
- **I_URC_P06** - Piano di monitoraggio ed esecuzione, verifica dei documenti originali
- **I_URC_P07** - TRIAL MASTER FILE, Investigator Site File e Archiviazione
- **I_URC_P08** - CRF e eCRF PER LA RACCOLTA DATI NEGLI STUDI CLINICI
- **I_URC_P09** - GESTIONE SISTEMI COMPUTERIZZATI
- **I_URC_P10** - GESTIONE SICUREZZA DEL PRODOTTO IN SPERIMENTAZIONE
- **I_URC_P11** - STESURA, REVISIONE E APPROVAZIONE DEL REPORT DELLO STUDIO CLINICO
- **I_URC_P13** - CONSENSO INFORMATO NELLE SPERIMENTAZIONI CLINICHE
- **I_URC_P14** - Stesura, revisione e approvazione del CLINICAL PROJECT MANAGEMENT PLAN
- **I_URC_P15** - Gestione operativa sperimentazioni cliniche
- **I_URC_P16** - Documentazione da inviare al CE e AC per l'approvazione del protocollo e/o eme
- **I_URC_P17** - DOCUMENTAZIONE DA INVIARE AL CET PER L'APPROVAZIONE DEL PROTOCOLLO
- **I_URC_P18** - GESTIONE DATI
- **I_URC_P20** - Gestione di serious breaches in studi clinici
